# Positional and Curvature Difference of Lamina Cribrosa According to the Baseline Intraocular Pressure in Primary Open-Angle Glaucoma: A Swept-Source Optical Coherence Tomography (SS-OCT) Study

**DOI:** 10.1371/journal.pone.0162182

**Published:** 2016-09-09

**Authors:** Yong Woo Kim, Jin Wook Jeoung, Michael J. A. Girard, Jean Martial Mari, Ki Ho Park

**Affiliations:** 1 Department of Ophthalmology, Armed Forces Capital Hospital, Seongnam, Korea; 2 Department of Ophthalmology, Seoul National University Hospital, Seoul National University College of Medicine, Seoul, Korea; 3 Department of Biomedical Engineering, National University of Singapore, Singapore, Singapore; 4 Singapore Eye Research Institute, Singapore, Singapore; 5 University of French Polynesia, Tahiti, French Polynesia; University of Melbourne, AUSTRALIA

## Abstract

**Purpose:**

To investigate the variation of lamina cribrosa (LC) structure based on the baseline intraocular pressure (IOP) in eyes with primary open-angle glaucoma (POAG) and healthy individuals using swept-source optical coherence tomography.

**Methods:**

A total of 108 eyes with POAG and 61 healthy eyes were recruited. Based on the baseline IOP, the POAG eyes were divided into higher-baseline IOP (HTG; baseline IOP > 21 mmHg, *n* = 38 eyes) and lower-baseline IOP (NTG; baseline IOP ≤ 21 mmHg, *n* = 70 eyes). The anterior laminar insertion depth (ALID), mean LC depth (mLCD), and the LC curvature index (mLCD–ALID) were measured, and compared among the three groups. The regional variation of LC structure was evaluated by vertical-horizontal ALID difference.

**Results:**

The mLCD and LC curvature index were greatest in HTG eyes (520.3 ± 123.0 and 80.9 ± 30.7 μm), followed by NTG (463.2 ± 110.5 and 64.5 ± 30.7 μm) and healthy eyes (382.9 ± 107.6 and 47.6 ± 25.7 μm, all *P* < 0.001). However, there were no significant difference in ALID between HTG and NTG eyes. The vertical-horizontal ALID difference was larger in NTG eyes (72.8 ± 56.2 μm) than in HTG (32.7 ± 61.4 μm, *P* = 0.004) and healthy eyes (25.5 ± 34.8 μm, *P* < 0.001).

**Conclusions:**

Lamina cribrosa position and curvature differed in POAG eyes with low and high IOP. This would support the theory that IOP induced biomechanical effects on the optic play a role on glaucoma.

## Introduction

The biomechanical theory suggests that the pathogenesis of glaucoma involves progressive axonal damage consequent upon IOP-related stress (force/cross sectional area) and strain (local deformation).[1, 2] It has been well documented by enhanced depth imaging spectral-domain optical coherence tomography (EDI SD-OCT) that the lamina cribrosa (LC) displaces posteriorly in glaucomatous eyes compared to healthy control.[3] In addition, our group recently demonstrated greater LC curvature in primary open-angle glaucoma (POAG) eyes compared to healthy control by using swept-source OCT.[4] However, axonal damage can occur, even within the normal range of IOP, if the optic nerve head (ONH) is susceptible to a given level of IOP-related stress/strain. Susceptibility to IOP is believed to be dependent on the morphology, the biomechanical properties, and the collagen fiber organization of ONH connective tissues (lamina and sclera).[5–8]

LC-structural comparison between high-tension glaucoma (HTG) and normal-tension glaucoma (NTG) is an attractive issue to be elucidated. Rho et al.[9] investigated LC depth between POAG (intraocular pressure [IOP] > 21 mmHg) and NTG (IOP < 21 mmHg) eyes by using time-domain OCT. Although there were no significant differences in LC depth between POAG and NTG eyes, the measurements in POAG eyes showed significant negative correlation with age. This kind of age-related difference in laminar displacement has been further confirmed by Ren et al.[10] The LC deformed less posteriorly in older eyes than younger eyes for a given level of visual-field loss. However, previous studies have considered how in-vivo LC structure, including its curvature and location of insertion, differs according to baseline IOP in human eyes.[4, 11]

The LC is known to show regional structural difference. Previous histological studies have reported that the superior and inferior regions of the LC have a larger pore size and fewer connective tissues than the nasal and temporal quadrants.[12–15] This regional variation of LC architecture has recently been confirmed by adaptive-optics SD-OCT.[16] This regional architectural difference can induce variable IOP-related stress for a given level of IOP, possibly leading to variable IOP-related strain by region. Building on this idea, our group recently reported increased vertical-horizontal peripheral LC depth (PLCD) difference in POAG eyes compared to healthy control.[11] This may imply that the peripheral LC in the vertical meridian might have increased IOP-related deformation compared with horizontal meridian in glaucomatous eyes. However, in that study the majority of the POAG population were NTG patients. Thus it is worth investigating regional variation in LC architecture for POAG patients with higher baseline IOP (HTG).

This would address the possibility that regional LC curvature[4] and vertical horizontal PLCD[11] differs between HTG and NTG eyes. The purpose of the present study was to investigate (1) the anterior laminar insertion depth (ALID), mean LC depth (mLCD), and LC curvature index differences among HTG, NTG and healthy eyes of similar age, (2) the vertical-horizontal ALID differences among the groups, and (3) the factors associated with greater vertical-horizontal ALID difference.

## Methods

### Study subjects

Written informed consent was obtained from each of the 169 subjects enrolled. The present study was approved by the Seoul National University Hospital Institutional Review Board and followed the tenets of the Declaration of Helsinki (1964).

We recruited 108 patients diagnosed with primary open-angle glaucoma (POAG) at Seoul National University Hospital Glaucoma Clinic and 61 healthy individuals of similar age who had visited the SNUH outpatient clinic for regular ocular check-ups (e.g., dry eye, cataract) and showed no abnormalities on disc stereophotography, red-free fundus photography, Cirrus HD-OCT, and standard automated perimetry. Participant recruitment was initiated from October 16, 2013. POAG was defined as including the presence of glaucomatous optic disc changes such as focal notching and thinning, RNFL defects on disc stereophotography and red-free fundus photography, glaucomatous VF defect, and an open angle confirmed by gonioscopic examination. Glaucomatous VF defect was defined as (1) glaucoma hemifield test values outside the normal limits or (2) three or more abnormal points with a probability of being normal of *P* < 5%, of which at least one point has a pattern deviation of *P* < 1%, or (3) a pattern standard deviation of *P* < 5%. The VF defects were confirmed on two consecutive reliable tests (fixation loss rate ≤ 20%, false-positive and false-negative error rates ≤ 25%).

The baseline IOP value was retrospectively chart-reviewed, and was defined as the mean of at least three measurements before initiation of IOP-lowering management. The baseline IOP for healthy individuals was defined as the mean of at least three IOP measurements from initial visits. Based on the baseline IOP values, the high-tension glaucoma (HTG) eyes were defined as POAG eyes with a baseline IOP > 21 mmHg, and normal-tension glaucoma (NTG) eyes were defined as POAG eyes with baseline IOP ≤ 21mmHg.[17, 18]

The subjects underwent a complete ophthalmic examination, including a visual acuity assessment, slit-lamp biomicroscopy, gonioscopy, Goldmann applanation tonometry, refractions, dilated fundus examination, disc stereophotography, and red-free fundus photography by digital fundus camera (VX-10, Tokyo, Japan) and standard automated perimetry (Humphrey C 30–2 SITA-Standard visual field; Carl Zeiss Meditec, Inc., Dublin, CA). The central corneal thickness (Pocket II; Quantel Medical, Clermont-Ferrand, France) and axial length (AXIS-II Ultrasonic Biometer; Quantel Medical S.A., Bozeman, MT) were measured. A 200×200 optic disc cube scan was performed by Cirrus HD-OCT (Carl-Zeiss Meditec), and the average peripapillary retinal nerve fiber layer thickness (RNFLT) and disc area were measured. The foveal-disc angle was measured from red-free fundus photograph, which was determined by the angle between the horizontal meridian through the center of optic disc and the axis connecting the fovea and the center of optic disc.

The present study excluded subjects with (1) a history of intraocular surgery including glaucoma surgery, (2) a history of intraocular disease (e.g., proliferative diabetic retinopathy, retinal vein occlusion, exudative age-related macular degeneration), (3) an axial length longer than 27 mm, (4) severely tilted optic discs, (5) OCT scans showing ambiguous visualization of the peripheral LC due to vascular shadowing or peripheral focal LC defects, or (6) less than three IOP measurements prior to IOP-lowering treatment.

### Swept-Source Optical Coherence Tomography Imaging of Optic Disc

All of the participants had been scanned with the DRI OCT-1 Atlantis 3D SS-OCT device (Topcon Medical Systems, Oakland, NJ). Five line cross scans (five lines horizontal and five lines vertical) centered at the optic disc and with 0.25-mm spacing between the cross-lines and a 6.0-mm scan width were performed. A total of 32 B-scans were averaged for each of the five cross-lines. The central three of the five cross-line scans (totals: 507 horizontal and 507 vertical scans of 169 subjects) were selected, and the mean of their measurements was used in the analysis. Of these, 31 scans (15 horizontal and 16 vertical scans) were excluded due to poor OCT scan quality that did not allow clear visualization of the peripheral LC (i.e., severe vascular shadowing). Therefore, a total of 492 horizontal scans and 491 vertical scans were included to the analysis. To enhance the visibility of the LC, adaptive compensation was applied to all of the optic disc scan images according to the relevant protocols (contrast exponent = 2, threshold exponent = 6).[19–21]

### Measurement of ALID, mLCD, and LC curvature index

All of the measurements were performed with ImageJ software (developed by Wayne Rasband, National Institutes of Health, Bethesda, MD; available at http://imagej.nih.gov/ij/), as described previously.[4]

The anterior laminar insertion depth (ALID) and mean LC depth (mLCD) were measured for LC position. The ALID was defined as the vertical distance between the anterior laminar insertion and the reference plane connecting the Bruch’s membrane openings (BMO). The mean values of the temporal and nasal ALID and those of the superior and inferior ALID were defined as the horizontal and vertical ALID, respectively. The area enclosed by the anterior laminar surface, the two vertical lines for the ALID measurement and the BMO reference plane was measured. The mLCD was computed by dividing this area by the length between the two vertical lines. The anterior LC surface was manually depicted as if there was no discontinuity on the anterior LC border, including vascular shadowing or LC pores. The horizontal and vertical mLCD were measured on each horizontal and vertical scan.

To evaluate the degree of posterior bowing, the horizontal and vertical LC curvature index measurements were defined as the difference between the mLCD and ALID (mLCD–ALID) on each horizontal and vertical scan, respectively. Thus, a higher LC curvature index indicated increased LC posterior bowing. The mean of the horizontal and vertical LC curvature index measures was defined as the overall LC curvature index.

To evaluate the regional variance of IOP-related structural change, the vertical-horizontal ALID difference was measured, since regional anatomical variance is more marked on the periphery[15], and numerous mathematical models predict that most IOP-induced LC deformation occurs in the peripheral LC.[2, 5–7, 22, 23]

An experienced ophthalmologist (Y.W.K.) blind to the subjects’ clinical information performed the measurements. Excellent intraobserver and interobserver reproducibility for the measurement of ALID and LC curvature index have been reported by Kim et al.[4]

### Statistical analysis

The continuous variables were compared among the three groups using one-way analysis of variance with Scheffe’s post hoc analysis. The categorical variables were compared using a chi-square test. The general linear model (GLM) was used to determine the factors (i.e. age, gender, diabetes mellitus, hypertension, baseline IOP, IOP at examination, central corneal thickness [CCT], axial length [AXL], average RNFLT, and mean deviation [MD] of VF) associated with greater vertical-horizontal ALID difference, first with a univariate model, and then with a multivariable model that included the univariate model variables with *P* < 0.10. Statistical analyses were performed with the Statistical Package for Social Sciences version 21.0 for Windows (SPSS, Inc., Chicago, IL). The level of statistical significance was set at *P* < 0.05. The data obtained are presented herein as mean ± standard deviation values.

## Results

### Baseline characteristics

The present study included 108 eyes of 108 POAG (38 HTG and 70 NTG) patients and 61 eyes of 61 healthy individuals of similar age (S1 Table). There were no differences in underlying disease (including diabetes mellitus and hypertension), refractions, axial lengths, disc area, or foveal-disc angle among the groups (Table 1). The NTG and healthy eyes had a higher proportion of female subjects than the HTG eyes. The baseline IOP was significantly higher in HTG eyes (24.3 ± 4.6 mmHg) than in NTG eyes (14.4 ± 3.1 mmHg, *P* < 0.001) or healthy eyes (13.6 ± 2.9 mmHg, *P* < 0.001). The IOP at examination was highest in HTG eyes (15.4 ± 3.0 mmHg), followed by healthy eyes (13.1 ± 2.7 mmHg) and NTG eyes (11.9 ± 2.0 mmHg, all *P* < 0.001). The CCT was significantly larger in HTG eyes (551.0 ± 31.1 μm) than in NTG eyes (526.7 ± 34.8 μm, *P* = 0.006). The average RNFLT, MD, PSD, and VFI values were significantly lower in glaucomatous eyes than in healthy eyes (all *P* < 0.001), but did not show any statistical differences between the HTG and NTG eyes (all *P* > 0.05). There were no significant difference in the average treatment period from the baseline examination to the time of OCT imaging between HTG (7.71 ± 5.55 years) and NTG (7.14 ± 4.89 years) groups (P = 0.71).

**Table 1:** Patient demographics.

	HTG (n = 38 eyes)	NTG (n = 70 eyes)	Healthy (n = 61 eyes)	P-value	Post hoc analysis
Age, year	54.7 ± 14.5	61.0 ± 10.1	60.7 ± 11.7	0.06*	
Female, *n* (%)	10 (26.3)	43 (61.4)	38 (62.3)	**0.001**†	
DM, *n* (%)	4 (10.5)	5 (7.1)	6 (9.8)	0.79†	
HTN, *n* (%)	9 (23.7)	21 (30.0)	19 (31.1)	0.71†	
Baseline IOP, mmHg	24.3 ± 4.6	14.4 ± 3.1	13.6 ± 2.9	< 0.001*	**A>B,C**
IOP at examination, mmHg	15.4 ± 3.0	11.9 ± 2.0	13.1 ± 2.7	< 0.001*	**A>C>B**
SE, D, (Range)	–1.9 ± 2.8 (–7.1, +2.1)	–1.0 ± 2.0 (–6.0, +3.0)	–0.7 ± 2.4 (–9.6, +2.8)	0.08*	
CCT, μm	551.4 ± 29.0	527.3 ± 31.8	538.6 ± 30.4	0.001*	**A>B**
AXL, mm	24.4 ± 1.3	24.2 ± 1.2	24.0 ± 1.3	0.23*	
Disc area, mm^2^	1.98 ± 0.36	2.02 ± 0.49	2.11 ± 0.38	0.26*	
Foveal-disc angle, °	7.06 ± 3.81	7.67 ± 3.47	7.15 ± 3.51	0.60*	
Average RNFLT, μm	66.7 ± 10.2	72.6 ± 11.0	90.3 ± 8.6	< 0.001*	**A,B<C**
MD, dB	–6.6 ± 6.8	–5.8 ± 3.8	–0.2 ± 1.2	< 0.001*	**A,B<C**
PSD, dB	7.0 ± 5.4	7.9 ± 4.2	1.7 ± 0.3	< 0.001*	**A,B>C**
VFI, %	82.2 ± 21.1	84.8 ± 13.1	99.7 ± 0.5	< 0.001*	**A,B<C**

Mean ± standard deviation, Statistically significant values are shown in bold.

* Comparison was performed using one-way analysis of variance with post hoc Scheffe’s multiple comparison testing,

^†^Comparison performed using chi-square test,

HTG = high-tension glaucoma, NTG = normal-tension glaucoma, DM = diabetes mellitus, HTN = hypertension, IOP = intraocular pressure, SE = spherical equivalents, CCT = central corneal thickness, AXL = axial length, RNFLT = retinal nerve fiber layer thickness, MD = mean deviation of visual field, PSD = pattern standard deviation, VFI = visual-field index, A = HTG, B = NTG, C = Healthy

### ALID, mLCD, and LC curvature index difference

The horizontal ALID was greatest in HTG eyes (423.1 ± 110.3 μm), followed by NTG eyes (362.2 ± 106.7 μm) and healthy eyes (322.6 ± 98.6 μm, all *P* < 0.001). The overall and vertical ALID was greater in HTG and NTG eyes than in healthy eyes, but did not show any statistical difference between the HTG and NTG eyes (Table 2).

**Table 2:** Comparison of LC structure in HTG, NTG and Healthy eyes.

	HTG (n = 38 eyes)	NTG (n = 70 eyes)	Healthy (n = 61 eyes)	P-value (*)	Post hoc analysis
ALID, μm					
Horizontal	423.1 ± 110.3	362.2 ± 106.7	322.6 ± 98.6	**< 0.001**	**A>B>C**
Vertical	455.8 ± 108.3	435.0 ± 110.2	348.1 ± 93.2	**< 0.001**	**A,B>C**
Overall	439.4 ± 104.9	398.6 ± 104.8	335.3 ± 94.4	**< 0.001**	**A,B>C**
Mean LC depth, μm					
Horizontal	515.6 ± 126.8	451.4 ± 114.1	385.0 ± 108.8	**< 0.001**	**A>B>C**
Vertical	525.1 ± 122.0	474.9 ± 111.4	380.8 ± 109.6	**< 0.001**	**A,B>C**
Overall	520.3 ± 123.0	463.2 ± 110.5	382.9 ± 107.6	**< 0.001**	**A>B>C**
LC curvature index, μm					
Horizonta1	92.5 ± 32.7	89.2 ± 39.6	62.4 ± 30.2	**< 0.001**	**A,B>C**
Vertical	69.3 ± 37.8	39.9 ± 36.3	32.8 ± 30.8	**< 0.001**	**A>B,C**
Overall	80.9 ± 30.7	64.5 ± 30.7	47.6 ± 25.7	**< 0.001**	**A>B>C**

Mean ± standard deviation, Statistically significant values are shown in bold.

* Comparison was performed using one-way analysis of variance with post hoc Scheffe’s multiple comparison testing.

HTG = high-tension glaucoma, NTG = normal-tension glaucoma, ALID = anterior laminar insertion depth, LC = lamina cribrosa, A = HTG, B = NTG, C = Healthy

The overall mLCD was greatest in HTG eyes (520.3 ± 123.0 μm), followed by NTG eyes (463.2 ± 110.5 μm) and healthy eyes (382.9 ± 107.6 μm, all *P* < 0.001). The HTG and NTG eyes showed a significant difference in horizontal mLCD but not in vertical mLCD (Table 2).

The overall LC curvature index was greatest in HTG eyes (80.9 ± 30.7 μm), followed by NTG eyes (64.5 ± 30.7 μm) and healthy eyes (47.6 ± 25.7 μm, all *P* < 0.001). The horizontal LC curvature index was significantly greater in HTG (92.5 ± 32.7 μm) and NTG (89.2 ± 39.6 μm) eyes than in healthy eyes (62.4 ± 30.2 μm, *P* < 0.001), but the vertical LC curvature index was increased only in HTG eyes (69.3 ± 37.8 μm) (*cf*. NTG [39.9 ± 36.3 μm] and healthy [32.8 ± 30.8 μm, *P* < 0.001] eyes).

**Fig 1:**
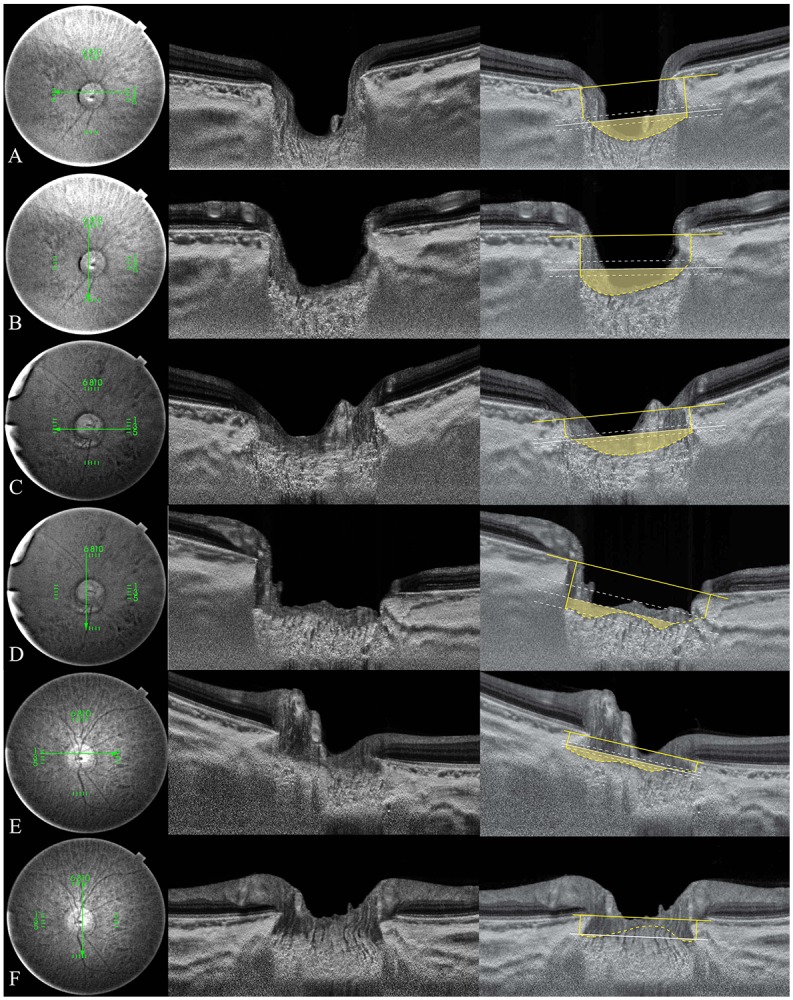
Representative swept-source optical coherence tomography (SS-OCT) B-scans of optic discs in high-tension glaucoma (HTG), normal-tension glaucoma (NTG), and healthy eyes. Horizontal (A, C, E) and vertical (B, D, F) optic disc scans of HTG (A, B), NTG (C, D) and healthy eye (E, F). The image delineated with yellow guidelines is the same as that depicted to the left. The area shaded with yellow depicts the degree of posterior bowing of the lamina cribrosa (LC) according to the level of anterior laminar insertion depth (white solid line). (A, B) Optic disc scans of 65-year-old male with primary open-angle glaucoma (POAG). His baseline intraocular pressure (IOP) was 45 mmHg, and his IOP at examination was 11 mmHg. The overall anterior laminar insertion depth (ALID) was 381.8 μm, the overall mean LC depth (mLCD) was 484.4 μm, and the overall LC curvature index was 102.7 μm. (C, D) Optic disc scans of 65-year-old male with POAG. His baseline IOP was 18 mmHg, and his IOP at examination was 13 mmHg. The ALID was 290.3 μm, the mLCD was 359.9 μm, and the overall LC curvature index was 69.6 μm. (E, F) Optic disc scans of healthy 46-year-old male. His IOP at examination was 13 mmHg. The ALID was 152.6 μm, the mLCD was 146.9 μm, and the overall LC curvature index was –5.7 μm.

Representative cases showing the ALID, mLCD, and LC curvature index differences among the groups are shown in Fig 1.

### Vertical-horizontal ALID difference

**Fig 2:**
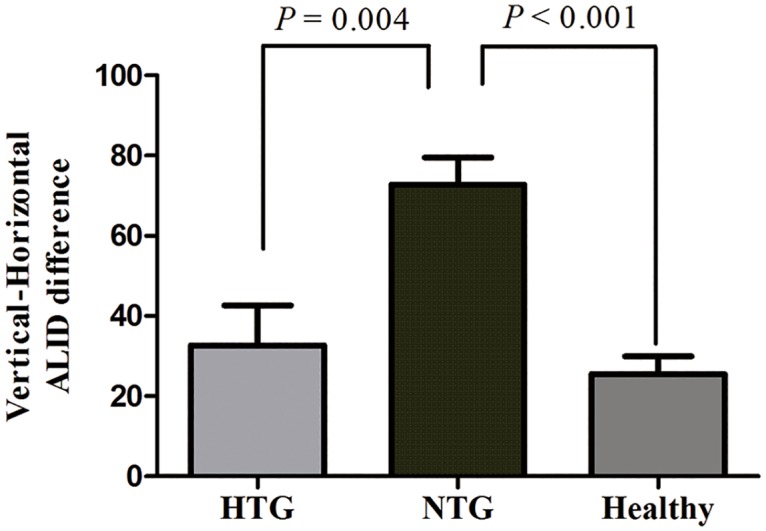
Vertical-horizontal ALID differences among HTG, NTG and healthy eyes. The vertical-horizontal ALID difference was significantly greater in NTG eyes (72.8 ± 56.2 μm) than in HTG (32.7 ± 61.4 μm, *P* = 0.004) or healthy (25.5 ± 34.8 μm, *P* < 0.001) eyes.

**Fig 3:**
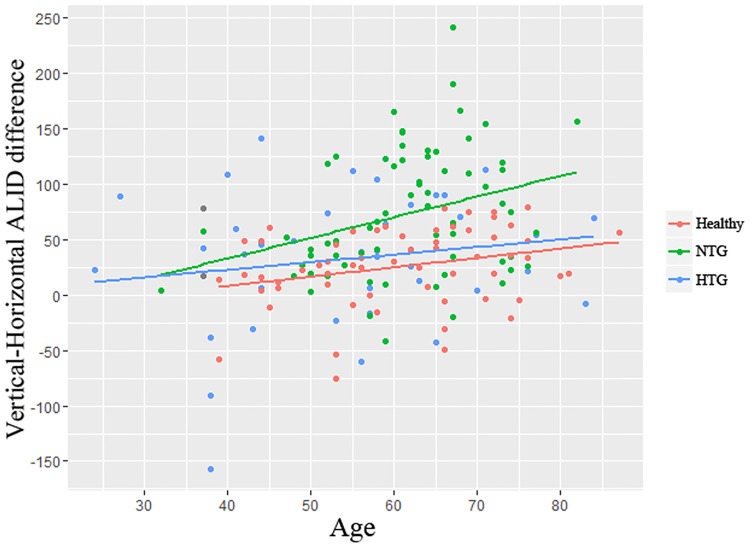
Relationship between age and vertical-horizontal ALID difference among HTG, NTG, and healthy eyes. The relationship between age and vertical-horizontal ALID difference is shown, with points colored according to their diagnosis. Regression lines are shown according to the diagnosis. The NTG eyes showed a greater age-dependent relationship than did the HTG or healthy eyes.

The vertical-horizontal ALID difference was significantly greater in NTG eyes (72.8 ± 56.2 μm) than in HTG (32.7 ± 61.4 μm, *P* = 0.004) or healthy (25.5 ± 34.8 μm, *P* < 0.001) eyes (Fig 2). The factors associated with greater vertical-horizontal ALID difference were investigated. In the univariate analysis, increased age (*P* = 0.001), presence of diabetes (*P* = 0.035), NTG diagnosis (*P* < 0.001), decreased baseline IOP (*P* = 0.025), and decreased average RNFLT (*P* = 0.031) were associated with greater vertical-horizontal ALID difference. The variables that showed significance at *P* < 0.10 (i.e., age, gender, diabetes, type of diagnosis, baseline IOP, IOP at examination, CCT, and average RNFLT) were included in the multivariable model. To avoid interactions between the type of diagnosis and baseline IOP or IOP at examination, the linear regression analysis was performed separately for these variables. In multivariable analysis model 1, age (beta = 0.878, *P* = 0.016) and NTG diagnosis (beta = 44.265, *P* < 0.001) was significantly associated with greater vertical-horizontal ALID difference. In multivariable analysis model 2, baseline IOP (beta = –2.835, *P* = 0.007) and average RNFLT (beta = –1.260, *P* < 0.001) was significantly associated with greater vertical-horizontal ALID difference (Table 3, Fig 3).

**Table 3:** Factors associated with increased vertical-horizontal ALID difference.

Variable	Univariate analysis			Multivariable analysis 1*			Multivariable analysis 2*		
	Beta	Standard error	P-value	Beta	Standard error	P-value	Beta	Standard error	P-value
Age, for each year older	**1.166**	**0.344**	**0.001**	**0.878**	**0.361**	**0.016**	0.614	0.369	0.10
Gender, male	–14.568	8.476	0.09	–4.917	8.568	0.57	–8.468	8.779	0.34
DM	**31.535**	**14.76**	**0.035**	26.210	13.781	0.06	27.733	14.358	0.06
HTN	15.640	9.314	0.11						
Diagnosis			**< 0.001**			**<0.001**			
HTG	7.197	10.546	0.33	11.402	14.776	0.44			
**NTG**	**47.299**	**9.000**	**< 0.001**	**44.265**	**11.338**	**<0.001**			
Healthy	Ref			Ref					
Baseline IOP, per 1 mmHg increase	**–1.741**	**0.771**	**0.025**				**–2.835**	**1.030**	**0.007**
IOP at examination, per 1 mmHg increase	–2.686	1.502	0.08				1.190	1.732	0.49
AXL, per 1 mm increase	–1.843	3.378	0.56						
CCT, per 1 μm increase	–0.249	0.133	0.09	–0.049	0.133	0.71	–0.025	0.139	0.86
MD of VF, per 1 dB increase	–0.858	2.066	0.41						
Average RNFLT, per 1 μm increase	**–0.809**	**0.299**	**0.031**	–0.140	0.420	0.74	**–1.260**	**0.345**	**<0.001**

Statistical analysis was performed using the general linear model. Statistically significant values are shown in bold,

* Factors with P < 0.10 in the univariate analysis were included in the multivariable analysis.

DM = diabetes mellitus; HTN = hypertension; HTG = high-tension glaucoma; NTG = normal-tension glaucoma; IOP = intraocular pressure; AXL = axial length; CCT = central corneal thickness; MD = mean deviation; VF = visual field; RNFLT = retinal nerve fiber layer thickness

## Discussion

The present study demonstrated that the LC located more posteriorly and showed greater curvature in POAG eyes relative to healthy eyes of similar age which was consistent with our previous findings.[4] Further, the data showed that the LC in HTG eyes located and bowed more posteriorly relative to that of NTG eyes. The vertical-horizontal ALID difference was greatest in NTG eyes, followed by HTG and healthy eyes.

**Fig 4:**
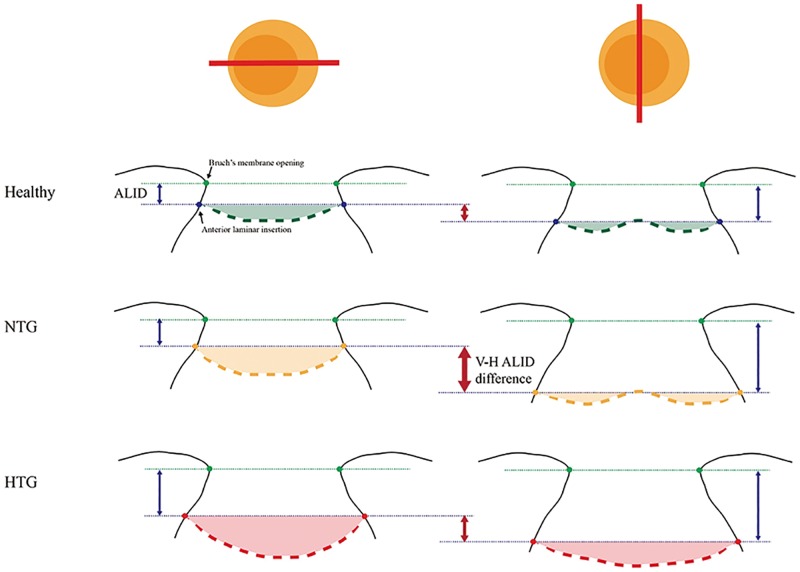
Schematic diagram showing LC-structural differences among HTG, NTG and healthy eyes. In healthy eyes, the anterior laminar insertion locates more posteriorly in superior and inferior axis compared to nasal and temporal axis, which results in vertical-horizontal ALID difference (small red arrow in the first line). In NTG eye, the vertical (superior-inferior) LC insertion locates much deeper than horizontal (nasal-temporal) LC insertion, which lead to increased vertical-horizontal ALID difference (large, thicker red arrow in the second line). The laminar curvature is increased in both meridians (yellow shaded area). In HTG eye, the horizontal anterior laminar insertion further locates posteriorly, which results in decreased vertical-horizontal ALID difference. The laminar curvature is much increased, so that the ‘w-shape’ contour changes to ‘u-shape’ contour in vertical meridian. The green points indicate Bruch’s membrane opening, and the thin green dotted-line corresponds to the reference plane connecting the BMO. The thicker green (healthy), yellow (NTG), and orange (HTG) dotted-lines indicate the anterior LC.

The horizontal ALID and mLCD measurement values were greater in HTG eyes than in NTG eyes, whereas the vertical ALID and mLCD measurements showed no significant difference between the two groups. Furthermore, the vertical-horizontal ALID difference was significantly greater in NTG eyes than in HTG eyes (Fig 4). The present observation might indicate that the LC in the vertical meridian is susceptible to IOP related stress/strain, even in cases of normal-range IOP, whereas the LC in the horizontal meridian becomes susceptible only at higher IOP. This discrepancy of the LC’s IOP susceptibility according to meridian (vertical or horizontal) might originate in the architectural difference between its superior and inferior and nasal and temporal areas.[12–15] The greater pore size and smaller number of connective tissues in the superior and inferior LC might compromise LC stability, subsequently leading to increased IOP-related strain for a given level of IOP. The present findings would seem to support the biomechanical theory of the relationship between baseline IOP and glaucoma pathogenesis.

LC posterior bowing (LC curvature index) was increased in HTG eyes relative to NTG eyes. Interestingly, the vertical LC curvature index was significantly greater in HTG eyes than in NTG eyes. This could have been due to the compressive effect of the LC’s central vessel trunk in HTG eyes. Park et al.[24] reported a good correspondence of central horizontal ridge with central vessel trunk position in healthy eyes. The curvature of the anterior LC surface in the vertical meridian, due to the presence of this central hump, therefore, appears to be “w-shaped.” Correspondingly, the vertical LC curvature index measurements were about half those of the horizontal LC curvature index in healthy eyes. The manifest increment of the vertical LC curvature index values in HTG eyes might indicate compression of the central vessel trunk. Contrastingly, the curvature of the vertical anterior LC surface from the central vessel trunk was somewhat maintained in NTG eyes, though the LC was displaced posteriorly in general (Fig 4). The position of the central vessel trunk, in fact, is known to be associated with glaucomatous neuroretinal rim change[25], parapapillary atrophy location[26], and the pattern of VF defects in glaucoma.[27] The influence of posterior displacement of central vessel trunk in HTG eyes on glaucoma severity or progression is beyond the scope of the present study, and calls for further evaluation through longitudinal assessment.

In the multivariable analysis, older age was significantly associated with increased vertical-horizontal ALID difference. We measured that difference to evaluate the regional variance of IOP-related LC-structural change. Our group previously reported increased vertical-horizontal peripheral LC depth (PLCD) difference in POAG eyes compared with healthy control eyes of similar age. This finding suggests increased IOP-related strain on the vertical meridian of the LC, where superior and inferior retinal nerve fiber layer defect commonly occurs.[11] In fact, the present data demonstrated an age-dependent relationship with vertical-horizontal ALID difference, especially in NTG eyes. The mechanical compliance of the LC is known to be diminished by age.[28] Ren et al.[10] demonstrated that the LC deformed less posteriorly in older eyes than in younger eyes for a given level of visual-field loss. Rho et al.[9] found a negative correlation between LC depth and age in POAG (HTG) eyes but not in NTG eyes. Taken together, LC response to IOP stress can vary not only by pressure intensity itself but also by age and region of LC.

The average RNFLT showed a significant negative association with the vertical-horizontal ALID difference. This finding is consistent with increased nerve fiber susceptibility to damage according to regional (vertical-horizontal) LC-structural difference in glaucomatous eyes. This finding, however, should be interpreted with caution, in that smaller vertical-horizontal ALID difference does not indicate intact RNFL status, since HTG eyes also showed smaller vertical-horizontal ALID difference. Certainly, given that HTG eyes manifested greater ALID, mLCD, and LC curvature index values relative to healthy eyes, vertical-horizontal ALID differences such as those identified in the present study should be interpreted in relation to other LC profile measurements.

The present study has the following limitations. First, the glaucoma patients were under intensive IOP-lowering treatments at the time of their enrollment. The baseline IOP was estimated, therefore, through retrospective chart-review, as a mean of at least three IOP measurements recorded prior to those IOP-lowering treatments. As such, the current baseline IOP data might not represent the subjects’ true diurnal IOP characteristics. Also, we cannot discount the influence of IOP-lowering treatments on LC structural change. LC depth is known to decrease after IOP-lowering treatment. Notwithstanding, we believe that our study population was relatively less affected, because both the HTG and NTG eyes had been exposed to a similar duration of maximally tolerable medical treatments, and also because we excluded eyes with a history of any glaucoma surgery. Further, well-controlled study with subjects naïve to IOP-lowering treatments and with diurnal IOP measurements could confirm our conclusion. Second, as the present study used only the central three cross-line B-scans with 0.25-mm spacing, the current data might not be representative of the entire LC architecture. Our data therefore should not be generalized to the superotemporal and inferotemporal regions of the LC, where RNFL defect most commonly occurs. Further assessment with radial optic disc scans would facilitate our understanding of the regional relationship between LC deformation and RNFL defect. Aditionally, the present data excluded eyes with peripheral focal LC defects, because continuous delineation of peripheral LC was essential for evaluation of ALID or the LC curvature index. However, such eyes have been reported to be correlated with localized RNFL defects[29] or disc hemorrhages.[30, 31] Thus, caution is needed so as not to generalize our conclusion to that eye type. Third, one may argue that the lack of FoBMO axis (the axis between the fovea and BMO center) correction may have biased the regionalization of LC when analyzing the vertical-horizontal LC parameters in the present study. It is proposed that the neuroretinal rim and peripapillary RNFL thickness parameters to be landmarked to the FoBMO axis to enhance the diagnostic performance and structure/function relationship.[32–34] However, the clinical relevance of this geometrical relationship between the fovea and the optic disc is still in controversy.[35, 36] To minimize this effect, we analyzed the foveal-disc angle and showed that there were no significant difference in HTG (7.06 ± 3.81°), NTG (7.67 ± 3.47°), and healthy eyes (7.15 ± 3.51°) (P = 0.60). In addition, the measurements of LC parameters have been performed in central three out of five cross-line B-scans. This ranges approximately 60° at each quadrant, which might have weakened the effect of discordance between the FoBMO and horizontal axes from the acquired image frame. Finally, the post-hoc power was only 15.6% in vertical ALID and 55.7% in vertical mLCD in the comparison between the HTG and NTG eyes. This result might have originated from the small study population, and moreover, it could have precluded the identification of significant differences between the two groups. For enhanced statistical power, further investigation into vertical structural difference in a larger study population should be conducted.

In conclusion, the present study demonstrated that the LC in HTG eyes locates more posteriorly and has greater curvature relative to NTG eyes and healthy eyes of similar age. NTG eyes showed greater vertical-horizontal ALID difference relative to HTG and healthy eyes. Difference in LC architecture according to IOP level seems to support the biomechanical theory of glaucoma pathogenesis.

## Supporting Information

S1 Table LC parameter measurement dataset. (XLSX)
